# The pill of recovery; Molnupiravir for treatment of COVID-19 patients; a systematic review

**DOI:** 10.1016/j.jsps.2022.03.002

**Published:** 2022-03-10

**Authors:** Lina Kamal, Ahmed Ramadan, Suha Farraj, Lydia Bahig, Sameera Ezzat

**Affiliations:** a Mounir Armanious Research Center (MARC), Egypt; b Department of Applied Statistics, Faculty of Postgraduate Studies for Statistical Research, Cairo University, Egypt; c Special Infectious Agents Unit, King Fahad Medical Research Center, King Abdulaziz University, Jeddah 21589, Saudi Arabia; d National Liver Institute, Menoufia University, Egypt

**Keywords:** Molnupiravir, COVID-19, Repurposing anti-viral drugs, NHC, EIDD-1931, MK-4482, EIDD-2807

## Abstract

**Background:**

Throughout the time of the global pandemic of SARS-CoV-2 virus, there has been a compelling necessity for the development of effective antiviral agents and prophylactic vaccines to limit the virus spread, disease burden, hospitalization, and mortality. Until mid of 2021, the NIH treatment guideline declared no single oral therapy was proven to treat mild to moderate cases. A new hope arose when a repurposed direct acting oral anti-viral agent “Molnupiravir” was shown to be effective in decreasing mortality and need for hospitalization in mild to moderate cases with relatively good safety profile; exhibiting a significant reduction in virus titers only after two days from administration. Molnupiravir recently granted the FDA emergency use authorization to treat mild to moderate COVID-19 patients with at least one risk factor for progression.

**Methods:**

We performed a computer-based literature search of (PubMed, Science direct, MedRxiv, BioRxiv, ClinicalTrials.gov, ISRCTN, Cochrane COVID study register, EU registry, and CTRI registry) till February 15th, 2022. The following keywords were used in our search (“Molnupiravir”, “NHC”, “EIDD-2807”, “MK-4482” or “EIDD-1931”).

**Results:**

We identified from the initial search a total of 279 articles; 246 articles (BioRxiv and MedRxiv N = 186, PubMed N = 33, Science direct N = 27) and 33 Clinical trials from the following registries (ISCTRN (N = 1), Clinical Trials.gov (N = 6), CTRI (N = 12), Cochrane (N = 14)). Through screening phases, 21 records were removed as duplicates and 198 irrelevant records were also excluded. The included studies in this systematic review were (N = 60) included 39 published papers and 21 clinical trials. After Manual addition (N = 4), the qualitative assessment included (N = 64).

**Conclusion:**

Based on the cumulative evidence from preclinical and clinical studies, Molnupiravir is proven to be a well tolerated, direct acting oral anti-viral agent to halt the disease progression in mild to moderate COVID-19 cases; in terms of mortality and hospitalization rates.

## 1 Introduction

As of February 27th, 2022, severe acute respiratory syndrome (SARS) coronavirus 2 (SARS-CoV-2), the virus responsible for the debilitating coronavirus disease-2019 (COVID-19), has caused more than 435,099,899 confirmed infections and 5,964,788 deaths worldwide (Worldometer, 2022). Both preclinical and clinical studies established association between SARS-CoV-2 RNA levels and the infectiousness of the host (Cox et al., 2021, Liu et al., 2020, Van Kampen et al., 2021, Wölfel et al., 2020, Bullard et al., 2020). Thus, the urgent need for oral antiviral therapies was revealed as effective antiviral therapies would in turn allow to reduce disease progression and halt virus transmission. Massive efforts have been placed into the innovation of newly developed anti-viral agents and protective vaccines, the repurposing of already available anti-viral drugs was the suitable direction to control the COVID-19 pandemic situation (Pardi and Weissman, 2020). There is an increasing list of antiviral agents extensively studied for their potential to be repurposed as an antiviral COVID-19 treatment (Milken Institute, 2022). To-date, ritonavir-boosted nirmatrelvir, molnupiravir, sotrovimab, remdesivir, chloroquine or hydroxychloroquine, lopinavir/ritonavir, ivermectin and other HIV protease inhibitors have been listed as anti-viral agents for treatment of COVID-19 at different stages of the disease (NIH, 2022). Indeed, few of any anti-viral regimens have been established to reasonably impact the major clinical outcomes such as disease progression (ICU admission and need for mechanical ventilation) or mortality. Previously, the WHO investigated four promising antiviral agents to be repurposed for treatment of COVID-19 infections. However, according to the last report published for the mega study, the four regimens had little or no effect on hospitalized patients with COVID-19, as indicated by overall mortality, initiation of ventilation, and duration of hospital stay (WHO Solidarity Trial Consortium, 2021). Similarly, agents like: sofosbuvir, favipiravir, oseltamivir, ribavirin, arbidol, nafamostat, nitazocanide and ivermectin were meticulously assessed as treatment options. However, most of these agents are still lacking established evidence to be appended to the list of approved antiviral agents (NIH, 2022).

Molnupiravir is the prodrug of the ribonucleoside analog β-D-N4-hydroxycytidine (NHC), which undergoes chemical conversion in plasma into NHC and then into active 5′-triphosphate form through host kinases (Painter et al., 2021). The active form acts as a substrate for virally-encoded RNA-dependent RNA polymerase (RdRp), causing its antiviral effect via increasing mutations that occur with each viral replication cycle (Agostini et al., 2019, Painter et al., 2019, 2021). Through a series of non-clinical studies, the drug exhibited antiviral activity against SARS-CoV-2 and other coronaviruses with a high barrier to resistance (Menachery et al., 2015, Sheahan et al., 2020, Wahl et al., 2021). Molnupiravir was repurposed for treating mild to moderate COVID-19 infection through an expedited clinical development program. On December 23, 2021, molnupiravir was granted the FDA Emergency Use Authorization (EUA) for the treatment of mild to moderate COVID-19 patients, who are at high risk of progressing to severe disease, when other antiviral therapies are not accessible or clinically appropriate. The drug was also listed in the NIH treatment guidelines for COVID-19. However, the drug is indicated only when other treatment options are not available for patients. The guideline panel preferred other antiviral therapies because they showed higher efficacy in terms of lower hospitalization/death rate in the treated groups vs placebo (NIH, 2022). Here, a systematic review on the cumulative evidence of molnupiravir safety and anti-viral activity is presented. Also, the review highlights a comparison between the current anti-viral agents listed to treat mild to moderate COVID-19 patients with risk for progression.

## 2 Methods

This systematic review complies with the preferred reporting items of the systematic review and meta-analysis (PRISMA) checklist (Moher et al., 2009). All stages were verified to be compliant with the Cochrane Handbook of Systematic Review and Meta-Analysis (Higgins et al., 2019). We performed a computer-based literature search of (PubMed, Science direct, MedRxiv, BioRxiv, ClinicalTrials.gov, ISRCTN, Cochrane COVID study register, EU registry, and Clinical Trial Registry-India (CTRI)) till February 15th, 2022. The following keywords were used in our search (“Molnupiravir”, “NHC”, “EIDD-2807”, “MK-4482” or “EIDD-1931”). Articles which assessed the safety and/or the anti-viral activity of Molnupiravir were included. Books, reviews, articles published before 2020, duplicate articles, or articles not written in English were excluded. Manual addition of articles was conducted on three consecutive steps: firstly, by tracking references of the retrieved studies. Secondly, by tracking the articles that cited the retrieved studies and lastly, we did a google search to retrieve the most recent news on Molnupiravir. Four independent authors (AR, LB, LK and SF) reviewed the literature search results according to the presented inclusion and exclusion criteria.

The retrieved records were transferred to a Mendeley shared library to detect and remove duplicate articles using “check duplicates” function. Citations were checked for their title, authors, journal, and year for manual removal of duplicate articles. Title and abstract screening were performed by four independent authors (AR, LB, LK and SF) using Mendeley citation manager according to the stated inclusion criteria. Any disagreement was judged and solved by the last author (SE). For the phase of full-text screening, all articles were downloaded and reviewed for their eligibility. Furthermore, the remaining references were exported to an Excel file with the key information for screening steps. The key information included: year of publication, authors, phase of assessment (preclinical and/or clinical), and main results. For the clinical trial registries, records were retrieved in CSV format when applicable, essential information of the clinical trials were retrieved and combined in one Excel sheet for further assessment. Clinical trials registered in multiple databases were manually removed. Finally, the decision to include or exclude articles for qualitative analysis had to be agreed upon by the four reviewers (AR, LB, LK and SF) to pass through. In case of any disagreements, the last author (SE) was requested to give a final decision.

## 3 Results

We identified from the initial search a total of 279 articles; 246 of them from databases (BioRxiv and MedRxiv N = 186, PubMed N = 33, Science direct N = 27) and 33 Clinical trials from the following registries (ISCTRN (N = 1), Clinical trials.gov (N = 6), CTRI (N = 12), Cochrane (N = 14)).

**Fig. 1:**
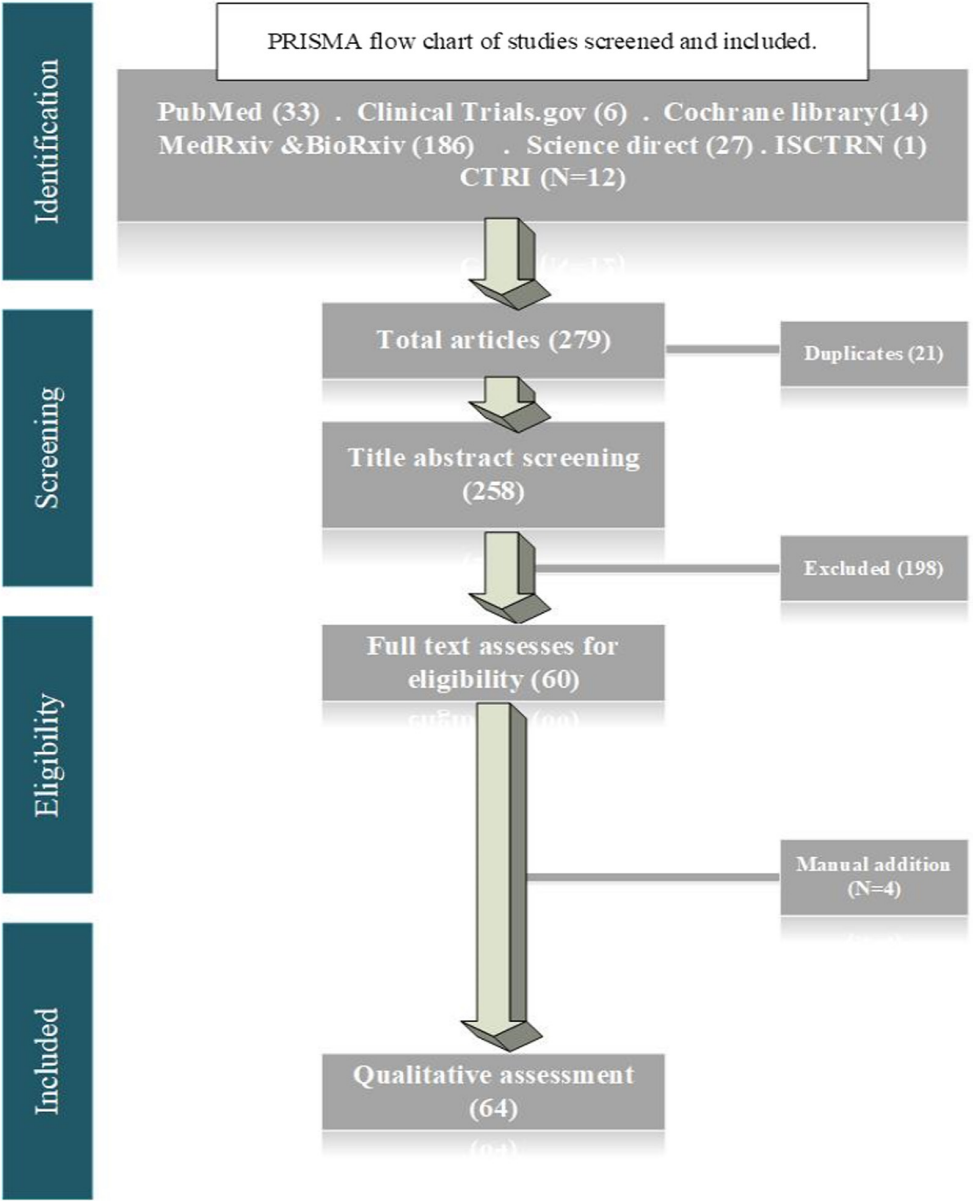
Prisma flowchart of the systematic review.

After reviewing abstracts, removing 21 records as duplicates, and excluding 198 irrelevant records, final included studies in this systematic review were (N = 60) including 39 published papers and 21 clinical trials. After manual addition of (Interim Clinical Results from Phase III Clinical Trials of Molnupiravir conducted in India + 3 more manual additions (N = 4)), Qualitative assessment included (N = 64) (Fig. 1. PRISMA flowchart).

## 4 The effect of Molnupiravir on SARS-CoV-2

The COVID-19 RNA-dependent RNA polymerase (RdRp) complex was synthetically produced in two separated studies with compatible results to investigate the mechanism of Molnupiravir-induced COVID-19 mutagenesis. This mechanism was set in a two-step model. The first step is when a new RNA strand synthesized using the original one. Although RdRp enzymes showed preferring natural nucleotides to synthesize the new strand, Molnupiravir nucleotide analogue (M) can act like C and base pairs with G or act like U and base pairs with A. This allows the synthesis strand smoothly extended with ambiguous nucleoside specially that the double strand RNA with analogue base pairs has approximately the same stability of the double strand with the natural base pairs. The second step of the mutagenesis model is when the RNA strand with Molnupiravir nucleotide analogue is used as template by RdRp enzymes. The resulted incorrect nucleotide can be one of three possibilities: 1 – If GTP incorporated with M, the incorporation of the next incoming nucleotide will be inhibited. Nonetheless, this inhibition can be overcome with the increasing of NTP concentrations, 2 – If ATP incorporated with M, it yields a G to A transition mutation via G:M:A, and 3 – If CTP incorporated with M, it yields a C to U transitions mutation via C:G:M:A:U. This explained the existence of the higher frequencies of G to A and C to U transition mutations with the using of Molnupiravir as an antiviral drug which consequently obstruct the replication of intact new viruses. This two-step model for the mechanism of Molnupiravir-induced coronavirus RNA mutagenesis like the suggested mutagenesis mode of action of Favipiravir but not the one of Remdesivir, which undertakes its action in the first step of the model by impairs RdRp progression (Gordon et al., 2021, Kabinger et al., 2021). The differences in the fighting mechanisms between these two antiviral drugs were supported by Sheahan et al. when they found that the error rate (no. of mutations/10,000 bases) is the same in treated viruses by remdesivir and untreated viruses whether the error rate was significantly increased in a dose-dependent manner when Molnupiravir was used (Sheahan et al., 2020). Wang et al. found that RdRp of SARS-CoV-2 can utilize nucleoside diphosphate (NDP) as a substrate in RNA syntheses as if it was a nucleoside triphosphate (NTP) with the same efficiency which is a unique feature of SARS-CoV-2 RdRP. Moreover, they approved that this fact also applied on β-d-N4-hydroxycytidine (NHC) diphosphate, called MDP, which used as a substrate by RdRp to be another active form of Molnupiravir as NHC triphosphate (MTP) (Wang et al., 2021a, Wang et al., 2021b).

The type of mutations produced by using Molnupiravir were studied computationally. Jena and her colleagues found that the binding between Molnupiravir nucleotide analogue and G base was more stable than the other analogues bases (Remdesivir, Galidesivir, Favipiravir, and Ribavirin) when they pair with normal bases. It is even more stable than the typical G:C pair and give similar structural to its Watson–Crick structure. This propriety can explain the escaping of exonuclease proofreading during the replication of the virus (Jena, 2020). This result confirmed using a SARS‐CoV replicon that lacks of exonuclease where Molnupiravir activity did not significantly affect (Lan et al., 2021). In another in-silico study, Padhi et al. found that the binding site in nonstructural protein 12 (nsp12) of SARS-CoV-2 is relatively immune to be mutant by Molnupiravir comparing to Remdesivir which can explain the effectiveness of Molnupiravir against Remdesivir-resistant SARS-CoV-2 (Padhi et al., 2021). A study for Agostini et al. MERS-CoV found that the predominant type of transition was lineage dependent which means it was G:A in one of the studied lineages where it had almost equal numbers of each transition type (G:A A:G and C:U and U:C) in another lineage of MERS-CoV. This study suggested that the accumulation of multiple transition mutations can achieve low-level resistance to Molnupiravir (Agostini et al., 2019).

Nucleotide transitions observed in MERS-CoV genomes when treated with Molnupiravir in vitro, were also observed in vivo. A positive correlation between the increasing of mutation rates and the frequency of codon change frequency, including stop codons and the degree of therapeutic efficacy in mice were found (Sheahan et al., 2020). In Abdelnabi et al. 2021 study done on SARS-CoV-2-infected hamsters, higher accumulation of mutations especially in C-to-T and G-to-A transitions were found in Molnupiravir treatment group in a dose dependent manner (Abdelnabi et al., 2021a).

## 5 The effect of Molnupiravir on host cell

Several preclinical studies measured the mutagenesis effect of using Molnupiravir on host DNA. Sheahan et al. didn’t find any accumulation of mutations when they tested the effect of Molnupiravir on mice lung tissues infected by MERS-CoV. They concluded that using Molnupiravir for virus treatment can’t incorporate into host cell DNA and thus can’t induce mutations in it because ribonucleotides are removed from eukaryotic cell DNA efficiently (Sheahan et al., 2020). A comparison between the mutagenicity of Molnupiravir, Favipiravir and Ribavirin was done in vitro in another study; Molnupiravir showed resistance in the studied colonies in a dose-dependent manner but did not inhibit cell growth. Favipiravir showed a modest yet significant increase in the number of resistant colonies but also without showing an inhibition in the cell growth. While ribavirin showed little to no resistant activity but inhibited cell growth. They suggested that the reason behind the resistant effect of Molnupiravir on the cell can be presumably caused by the conversion to its intermediate DNA precursor that produced by normal metabolic pathway (Zhou et al., 2021a).

Molnupiravir antioxidant properties were studied by Martínez. In general, free radicals can be hunted by electron donor or electron acceptor molecules and thus oxidative stress can be prevented. Most antiviral drugs like Remdesivir and Ribavirin are electron donors which can affect viral infection by reducing molecules necessary for essential steps in its life cycle. This can also be a side effect for the drug because they reduce other essential molecules for patient life. The same principal can be applied if the antiviral drug was an electron acceptor which can oxidize molecules that are essential for both virus and human. Molnupiravir is an electron acceptor but not a good one, which means it is not an effective oxidant. Therefore, its side effects can be minimal from this viewpoint when it's used against COVID-19 (Martínez, 2021).

## 6 The activity of Molnupiravir against SARS-CoV-2 variant of concern

A number of in vitro and in vivo studies were done to investigate the activity of Molnupiravir against different SARS-CoV-2 variant of concern (VoC), involving alpha (B.1.1.7), beta (B.1.351), gamma (P.1.), delta (B.1.617.2), omicron (B.1.1.529) and the origin lineage A (Wuhan strain). These studies agreed that the inhibition of the virus by Molnupiravir was similar between all VoC (Abdelnabi et al., 2021a, Bojkova et al., 2022, Lieber et al., 2022, Prince et al., 2021, Rosales et al., 2022, Vangeel et al., 2022). In one of these studies, the result of this reduction remained equivalent when Human ACE-2 A549 cells were treated with Molnupiravir prior-infection, at the infection, after two, four- or 24-hours post-infection. When the treatment was given after two days from the infection, the activity of the drug was reduced for all tested CoV which could justify why the use of Molnupiravir was stopped in hospitalized patients and just licensed for the mild-moderate outpatients (Prince et al., 2021). In Syrian hamsters, there was also a comparable significant improvement in the pathology of lung with different VoC infections (Abdelnabi et al., 2021a). Also, they found in ferrets models a consistent of VOC shedding from the upper respiratory which avoided viral transmission. However, unequal shedding was found in the lungs of treated dwarf hamsters which was VOC-dependent although all the animals were completely cured regardless the injected VoC. Surprisingly, Omicron showed sex-dependent response to treatment in which the reduction in males was overall better than females without seeing this difference with VOC gamma or delta (Lieber et al., 2022).

The similarity of viral load reduction between the different VoC of SARS-CoV-2 when treated with Molnupiravir was expected because the drug is targeting the replication process of the virus which depends on highly conserved viral protein with minimal missense mutations (Bojkova et al., 2022, Vangeel et al., 2022).

## 7 Combination of antiviral drug with Molnupiravir to inhibit SARS-CoV-2

A combination of Molnupiravir and Favipiravir was obtained by Syrian hamsters directly before infecting them with SARS-CoV-2. A higher reduction of viral titer was observed for the combo-treatment compared to mono-treatment. Abdelnabi *et al.* explained this marked antiviral effect by the increasing of mutation accumulation count in the viruses treated with the combo dosage regarding the single treatment, especially for C-to-T mutations. This superiority also appeared when the treatment was delayed until six or 24 h post infections. Worth to mention, when the treated infected hamsters co-housed with healthy ones, approximately no transmission of virus was observed, hoping that the combo-treatment of infected patients will largely reduce the possibility of transmission (Abdelnabi et al., 2021b). In another experiment, Wang et al. reported the using of Pibrentasvir (hepatitis C virus NS5A inhibitors) as SARS-CoV-2 exonuclease inhibitors. Through a combination with Molnupiravir, Pibrentasvir largely protects the nucleotide analogues derived from Molnupiravir from being excised by the virus exonuclease from the 3′ terminus of RNA comparing with the absence of Pibrentasvir in which there was a rapid excision (Wang et al., 2021a, Wang et al., 2021b). Jonsdottir et al. tested a combination of Molnupiravir with different drugs. The combination with Ivermectin, camostat apilimod, alisporivir, nafamostat, or brequinar showed significantly higher antiviral activity in the qPCR analysis comparing with the individual use of Molnupiravir or any of these drugs. However, that wasn’t the result with remdesivir, ONO-3307 or TO-195 combinations. Overall, all the combinations presented no detectable infectious virus at 72 h prior infection (Jonsdottir et al., 2022).

## 8 Molnupiravir in clinical trials

Phase 1 studies on molnupiravir were conducted when preliminary data were submitted on the activity of the promising drug. In a study testing human safety, tolerability, and Pharmacokinetics of Molnupiravir, Molnupiravir was well absorbed in doses from 50 to 1600 mg, and the parent Ribonucleoside analogue EID-1931 was detected in plasma in a linear, dose proportional manner. It was also observed that absorption rate is lower in the fed state and took longer time (lower Tmax and Cmax); yet the extent of absorption (AUC inf.) wasn’t significantly different between fed state and fasted state. Molnupiravir showed safety and tolerability at a range from 50 to 800 mg dose when taken twice daily for 5–5.5 days or when taken as single dose up to 1600 mg in several trials. There was no accumulation reported in the multiple ascending dose group; a trace amount of Molnupiravir was found in urine due to EID-1931 metabolism by the kidneys into Cytidine and Uridine. The drug was found to be well tolerated and the reported adverse events (AEs) were mild, non-serious, rapidly resolved and didn’t cause drug discontinuation. The most common observed AEs reported were headache with the single ascending dose, diarrhea in the multiple ascending doses, also those AEs were reported in placebo group; headache (18.8% placebo in comparison to 12.5% Molnupiravir) and diarrhea (7.1% in both placebo and Molnupiravir). Also, there were no serious adverse events (SAEs) or dose-related events in the labs, clinical assessment, vital signs, and ECG of subjects (Painter et al., 2021). In another Phase 1 controlled trial [NCT04392219] on healthy volunteers, subjects were administered doses of 300, 600 and 800 mg of Molnupiravir. Twice daily dose of 800 mg of Molnupiravir was found to be safe and tolerable in participants with SARS CoV-2 infection in the AGILE trial [NCT04746183] (Khoo et al., 2021). All AEs reported were mild (≤grade 2 severity); diarrhea, nausea, cough, loss of smell or taste and flu like symptoms were reported and rapidly resolved. Hence, A remarkable advantage of Molnupiravir over other injectable antiviral drugs is its oral availability, possibility of widespread global application as well as a favorable safety profile and high tolerability (Holman et al., 2021, Rosenke et al., 2021).

Molnupiravir rapidly preceded into phase II trials. Phase IIa study [NCT04405570] investigated Molnupiravir in the treatment of participants with SARS-CoV-2 infection who showed significant viral load reduction at day 5 from administration with significantly higher reduction in the 800 mg dose-group. Molnupiravir antiviral efficacy was verified upon elimination of virus from nasopharyngeal swabs of participants who received the 400 or 800 mg dose showing median viral RNA change from baseline on days 3, 5, 7, 14, and 28. At day 3, 1.9% of the 800 mg dose group detected the virus in swabs while 16.7% of the placebo group showed viral detection. Complete viral elimination from swabs was achieved in the 400 & 800 mg dose group at day 5 while 11.1% of placebo still isolated the virus, (P = 0.034 and 0.027, respectively). Viral RNA clearance in the 800 mg dose-group was successfully achieved with percentage of 92.5% of participants in comparison to 80.3% in placebo till the end of study (day 28). Despite this study’s limitation which is the mis proportion in randomization between seropositive and low viral load in participants receiving the 800 mg dose, the study showed compatible results with the interim analysis published from a phase 3 clinical trial on mild to moderate COVID-19 patients. Both studies showed significant reduction in the hospitalization time and death in patients treated with the 800 mg dose. Also, risk of death decreased by 89% in patients who received Molnupiravir (Fischer et al., 2022).

As of 15th of February 2022, Molnupiravir was being assessed in 21 clinical trials (Table 1).

**Table 1:** List of clinical trials assessing safety and efficacy of Molnupiravir for treatment of COVID-19.

Rank	ID number	Title	Status	Phase	Size	Interventions	Study design
*Clinical trials.gov (N = 6)*							
1	NCT04575584/Other Study identifier on Cochrane (JRCT2031200404)	Efficacy and Safety of Molnupiravir (MK-4482) in Hospitalized Adult Participants With COVID-19 (MK-4482–001)	Terminated	Phase 2/Phase 3	1300	Drug: Molnupiravir administered orally in capsule form every 12 h for 5 days (10 doses total) Placebo matching Molnupiravir administered orally in capsule form every 12 h for 5 days (10 doses total)	• Allocation: Randomized Intervention Model: Parallel Assignment Masking: Double (Participant, Investigator) Primary Purpose: Treatment
2	NCT04575597/Other Study Identifier on Cochrane (JRCT2031210148)	(MOVe-OUT) Efficacy and Safety of Molnupiravir (MK-4482) in Non-Hospitalized Adult Participants With COVID-19 (MK-4482-002)	Completed	Phase 2/Phase 3	1850	Drug: Molnupiravir | Drug: Placebo	• Allocation: Randomized Intervention Model: Parallel Assignment Masking: Double (Participant, Investigator) Primary Purpose: Treatment
3	NCT04939428	(MOVe-AHEAD) Study of MK-4482 for Prevention of Coronavirus Disease 2019 (COVID-19) in Adults (MK-4482-013)	Recruiting	Phase 3	1332	Drug: Molnupiravir/|Drug: Placebo	• Allocation: Randomized Intervention Model: Parallel Assignment Masking: Triple (Participant, Investigator, Outcomes Assessor) Primary Purpose: Prevention
4	NCT04405739	The Safety of Molnupiravir (EIDD-2801) and Its Effect on Viral Shedding of SARS-CoV-2 (END-COVID)	Recruiting	Phase 2	96	Drug: EIDD-2801/Drug: Placebo	• Allocation: Randomized Intervention Model: Parallel Assignment Masking: Double (Participant, Investigator Primary Purpose: Treatment
5	NCT04405570/Other identifier on PubMed (PMID: 35022711)	A Safety, Tolerability and Efficacy of Molnupiravir (EIDD-2801) to Eliminate Infectious Virus Detection in Persons With COVID-19	Completed	Phase 2a	204	• Initially, participants were randomized in a 1:1 ratio to receive 200 mg molnupiravir or placebo twice daily for 5 days. Subsequently, in the dose-range finding portion of the study, participants were randomized in a 3:1 ratio to receive 200, 400, or 800 mg molnupiravir or placebo twice daily for 5 days.	• Allocation: Randomized Intervention Model: Cross-Sectional Masking: Double blinded (Participant, Investigator) Primary Purpose: Treatment
6	NCT04746183/Other identifier on Cochrane (EUCTR2020-001860-27)	AGILE (Early Phase Platform Trial for COVID-19)/AGILE: seamless Phase I/IIa Platform for the Rapid Evaluation of Candidates for COVID-19 treatment. A randomized Phase I/II study to determine the safety and effectiveness of multiple drugs for the treatment of COVID-19	Recruiting	Phase 1|Phase 2	600	Drug: CST-2: EIDD-2801|Drug: CST-2: Placebo| Drug: Nitazoxanide| Drug: VIR-7832|Drug: VIR-7831|Drug: CST-5: Placebo INTERVENTION: Phase I – Patients randomized to EIDD-2801 or standard of care (SOC). EIDD-2801 administered orally, twice daily (BID) for 10 doses (5 or 6 days). Phase II – Patients randomized to EIDD-2801 and SOC or Placebo and SOC. EIDD-2801 or placebo administered orally, twice daily (BID) for 10 doses (5 or 6 days). Dose of EIDD-2801 will be determined by the recommended dose from Phase I. Patients randomized 1:1 between EIDD-2801 and placebo using permuted block randomization stratified by site	• Allocation: Randomized Intervention Model: Sequential Assignment Masking: Quadruple (Participant, Care Provider, Investigator, Outcomes Assessor) Primary Purpose: Treatment

*Clinical Trials Registry of India (CTRI) (N = 12)*							
7	CTRI/2021/05/033693	A prospective, randomized, parallel, multicentric, phase-III clinical trial of Molnupiravir 800 mg capsules and standard of care (SOC) compared to standard of care only in confirmed RT-PCR positive patients with mild COVID-19.	Recruiting	Phase 3	1218	• Molnupiravir 200 mg capsules, (4 × 200 mg), twice a day for 5 days before food intake Standard of Care (Ivermectin 12 mg, oral tablet, once daily for 5 days after food intake. Symptomatic medication including anti-pyretic, anti-tussive and multivitamins, Empiric antimicrobial)	• Allocation: Randomized Intervention Model: Parallel Assignment Masking: Open label
8	CTRI/2021/05/033739	A Phase III, Multicentric, Prospective, Randomized, Parallel Study to Evaluate the Efficacy and Safety of Molnupiravir in Adult Indian Patients with Mild COVID-19	Not yet recruiting	Phase 3	1218	• Molnupiravir 800 mg (4 capsules of 200 mg) administered orally every 12 h for 5 days AND Standard of Care Comparator Agent: Standard of Care as per the ICMR Clinical Management Protocol	• Allocation: Randomized Intervention Model: Parallel Assignment Masking: Open label
9	CTRI/2021/05/033736	A Phase III, Multicentric, Prospective, Randomized, Parallel Study to Evaluate the Efficacy and Safety of Molnupiravir in Adult Indian Patients with Moderate COVID 19	Not yet Recruiting	Phase 2/Phase 3	1282	• Molnupiravir 800 mg (4 capsules of 200 mg) administered orally every 12 h for 5 days (10 doses total) plus Standard of Care. Comparator Agent: Standard of care as per the Clinical Guidance for Management of Adult COVID-19 by ICMR.	• Allocation: Randomized Intervention Model: Parallel Assignment Masking: Open label
10	CTRI/2021/05/033904	A Prospective, Randomized, Parallel, Multi-centric, Open label, Phase III Clinical Trial to Evaluate the Efficacy and Safety of Molnupiravir Capsule in Treatment of Subjects with Mild Corona virus Disease (COVID-19)	Recruiting	Phase 3	1218	• Molnupiravir: 4 capsules of 200 mg Twice daily for 5 days Comparator Agent: Standard of Care treatment shall be based on the Clinical Guidance for Management of Adult COVID-19 Patients, dated 22 Apr 2021. AIIMS/ ICMR-COVID-19 National Task Force/Joint Monitoring Group (Dte. GHS) and recommendations of Ministry of Health and Family Welfare, Government of India.	• Allocation: Randomized Intervention Model: Parallel Assignment Masking: Open label
11	CTRI/2021/06/034130 / Same identifier on Cochrane	A Multi-Centric,Prospective, open label, Randomized, Parallel-group, Comparative, Phase III Clinical Trial to evaluate the efficacy and safety of Molnupiravir 800 mg in the treatment of patients diagnosed with mild COVID-19	Recruiting	Phase 3	1218	• 4 capsules of Molnupiravir 200 mg each will be given twice a day at interval of 12 h Comparator Agent: Ivermectin, symptomatic medication including oral hydration, anti-pyretic, anti-tussive and multivitamins, Empiric antimicrobials	• Allocation: Randomized Intervention Model: Parallel Assignment Masking: Open label
12	CTRI/2021/05/033864	A Prospective, Randomized, Parallel, Multi-centric, Open label, Phase III Clinical Trial to Evaluate the Efficacy and Safety of Molnupiravir Capsule in Treatment of Subjects with Moderate Coronavirus Disease (COVID-19)	Recruiting	Phase 3	1282	• Molnupiravir 800 mg (4 capsules of 200 mg) administered orally every 12 h for 5 days AND Standard of Care Comparator Agent: Standard of care as per the Clinical Guidance for Management of Adult COVID-19 by ICMR	• Allocation: Randomized Intervention Model: Parallel Assignment Masking: Open label
13	CTRI/2021/06/033938	A Prospective, Randomized, Multicenter, Open Label, Parallel Group Study To Evaluate Safety And Efficacy Of Oral Molnupiravir As Add On To Standard Of Care For Treatment Of Mild Patients With Covid-19 Disease	Recruiting	Phase 3	1218	• Molnupiravir Tablets 200 mg (4 × 200 mg), twice a day for 5 days before food intake Comparator Agent: Standard of Care	• Allocation: Randomized Intervention Model: Parallel Assignment Masking: Open label
14	CTRI/2021/06/033992	A prospective, randomized, parallel, multicentric, phase III clinical trial to assess the efficacy and safety of Molnupiravir 800 mg capsules and standard of care (soc) compared to standard of care (soc) only in patients with polymerase chain reaction (RT-PCR) confirmed Mild Covid-19 infection.	Closed Recruitment	Phase 3	1218	• Molnupiravir 800 mg (4x200 mg or 2x400 mg) capsules twice a day (BID) for 5 days plus standard of care (SOC) Comparator Agent:Standard of Care (SOC) only	• Allocation: Randomized Intervention Model: Parallel Assignment Masking: NA
15	CTRI/2021/06/034220	A Multi-Centric, Prospective, open label, Randomized, Parallel-group, Comparative, Phase II/III Clinical Trial to evaluate the efficacy and safety of Molnupiravir 800 mg in the treatment of patients diagnosed with moderate COVID-19.	Recruiting	Phase 2/Phase 3	1282	• Molnupiravir 200 mg capsules, (4 × 200 mg), twice a day,2 capsules of 200 mg each will be given twice a day at interval of 12 h Comparator Agent: Standard care of therapy: Oxygen therapy through non-rebreathing face mask, Anti-inflammatory or immunomodulatory therapy: Inj. Methylprednisolone 0.5 to 1 mg/kg in 2 divided doses (or an equivalent dose of dexamethasone) usually for a duration of 5 to 10 days. Patients may be initiated or switched to oral route if stable and/or improving, Anticoagulation: Conventional dose prophylactic unfractionated heparin or Low Molecular Weight Heparin (weight based e.g., enoxaparin 0.5 mg/kg per day SC) Symptomatic management (oral hydration, anti-pyretics, antitussive, multivitamins),Empiric antimicrobials for co-infections	• Allocation: Randomized Intervention Model: Parallel Assignment Masking: Open label
16	CTRI/2021/06/034015	A Multi-Centre, Prospective, Open Label, Parallel, Randomized, Clinical Trial to Assess the Efficacy And Safety Of Molnupiravir 800 Mg Capsules And Standard of Care (SoC) Compared To Standard of Care (SoC) Only In Mild Patients With Polymerase Chain Reaction (PCR) Confirmed COVID-19.	Recruiting	Phase 3	1220	• Molnupiravir 800 mg (4 capsules of 200 mg or 2 capsules of 400 mg) (BID) + Standard of care. Patients will be instructed to take Comparator Agent: The standard of care will be as per physician recommendation or prescription in line to -Revised Guidelines on Clinical Management of COVID-19 by Government of India, Ministry of Health & Family Welfare Directorate General of Health Services, (EMR Division), Version 05, 03rd July 2020.. Treatment may include Oral medications like Ivermectin 12 mg, once daily, anti-pyretic, anti-tussive multivitamins, and antibiotics	• Allocation: Randomized Intervention Model: Parallel Assignment Masking: Open label
17	CTRI/2021/07/034588	A Phase 3 Prospective Open Label, Randomized Multicenter Parallel Study to evaluate the efficacy and safety of Molnupiravir capsules when administered along with Standard of Care compared to Standard of Care alone in Indian patients with mild COVID-19 disease	Completed	Phase 3	1220	• Molnupiravir plus standard of care and standard of care alone As per ICMR. Duration of Treatment: 5 days oral twice daily (800 mg) Comparator Agent: Standard of care therapy will be given to the subjects as per the institution practice Duration of Treatment: 5 days	• Allocation: Randomized Intervention Model: Parallel Assignment Masking: Open label
18	CTRI/2021/08/035424	A Phase 3 Prospective, Open Label, Randomized, Multicenter, Parallel Study to evaluate the efficacy and safety of Molnupiravir capsules when administered along with Standard of Care compared to Standard of Care alone in Indian patients with Moderate COVID-19 disease	Not Yet Recruiting	Phase 3	100	• Molnupiravir: 5 days treatment with 1600mgs oral twice daily Comparator Agent: Standard of care therapy will be given to the subjects as per the institution practice	• Allocation: Randomized Intervention Model: Parallel Assignment Masking: Open label

*Cochrane (N = 3)*							
19	JPRN-JRCT2031210010	Single and Multiple Dose Study of MK-4482 in Healthy Japanese Adults	Not yet recruiting	Phase I	72	• INTERVENTION: - Drug: MK-4482 (molnupiravir) MK-4482 100–1600 mg administered orally in capsule form once or twice daily (every 12 h for 5.5 days, 11 doses in total) Placebo matching MK-4482 administered orally in capsule form once or twice daily (every 12 h for 5.5 days, 11 doses in total)	Randomized, Placebo-Controlled, Double-Blind
20	NCT04392219	A Randomized, Double-Blind, Placebo-Controlled, First-in-Human Study Designed to Evaluate the Safety, Tolerability, and Pharmacokinetics of EIDD-2801 Following Oral Administration to Healthy Volunteers	Completed	Phase I	130	Drug: EIDD-2801 Part 1: Subjects will be randomized to receive a single oral dose of EIDD-2801 or Placebo. Part 2: Two single oral doses of EIDD-2801 will be administered to subjects, in an open-label manner. Part 3: Subjects will be randomized to receive twice daily dosing either EIDD-2801 or Placebo. Drug: Placebo Part 1: Subjects will be randomized to receive a single oral dose of EIDD-2801 or Placebo. Part 3: Subjects will be randomized to receive twice daily dosing either EIDD-2801 or Placebo.	Randomized, Placebo-Controlled, Double-Blind
21	NCT05195060	TURN-COVID Biobank: The Dutch Cohort Study for the Evaluation of the Use of Neutralizing Monoclonal Antibodies and Other Antiviral Agents Against SARS-CoV-2 (TURN-COVID)	Recruiting	Observational	1000	Drug: casirivimab with imdevimab Monoclonal antibody Drug: sotrovimab Monoclonal antibody Drug: molnupiravir Antiviral agent	• Observational Model: Cohort Time Perspective: Prospective Primary Purpose: Therapeutic effect and Cost-effectiveness

Of them, six are registered on clinical trial.gov database. Two of the six trials are Phase II aiming to assess the safety of Molnupiravir and its effect on viral shedding of SARS-CoV-2 and on eliminating infectious virus detection in persons with COVID-19 [NCT04405739 and NCT04405570, respectively]. Most importantly, there were three Phase III trials on Molnupiravir; one of which is currently ongoing for assessing the potential prevention of COVID-19 [MOVe-AHEAD, NCT04939428], one is terminated [NCT04575584], and one is completed [MOVe-OUT, NCT04575597].

The FDA emergency use authorization of Molnupiravir was based on the results of MOVe-OUT study. In this study, the treatment effect estimates for the primary end point, which was the rate of hospitalization or death through day 29, was reduced in the all-randomized analysis as compared with the interim analysis [approximately 30 % vs 50%]. Although Molnupiravir showed a significant mortality benefit; a risk of death was lower by 89% (95% CI, 14–99%) with Molnupiravir than with placebo. And the investigators considered all the deaths to be COVID-19–related (Table 2). Also, MOVe-OUT study showed WHO Clinical Progression Scale benefits with molnupiravir over placebo (Jayk Bernal et al., 2021). The difference between the interim and the all-randomized analyses’ results are summarized in Table 2. Many factors were likely to be correlated with this difference. Of them, there were more participants with low viral load at baseline (in whom there is less virologic effect) at the time of the all-randomized sample than at the time of the interim analysis. In addition, newly enrolling countries, were included in all-randomized sample which may also have affected hospitalization rates as part of the primary outcome.

**Table 2:** Differences between Interim analysis and all randomized sample analysis for MOVe-OUT trial.

Parameters		Interim analysis N = 775		All Randomized sample N = 1433	
Study groups		Molnupiravir N = 387	Placebo N = 388	Molnupiravir N = 716	Placebo N = 717
Baseline demographic & clinical characteristic		Almost similar	Almost similar	Almost similar	Almost similar
Gender	Female, no. (%)	200 (51.7)	171 (44.1)	384 (53.6)	351 (49.0)
	Male, no. (%)	187 (48.3)	217 (55.9)	332 (46.4)	366 (51.0)

Risk of hospitalization or death through day 29 no. (%)		28 (7.3)	53 (14.1)	48 (6.8)	68 (9.7)
Death		0 (0)	8 (2)	1 (0.1)	9 (1.3)
Incidence of any AE (%)		35%	40%	30.40%	33%
Incidence of drug-related AEs (%)		12%	11%	8.00%	8.40%

The CTRI registry had 12 trials registered as phase III trials with total number of 13,694 patients. Other Japanese trials followed the same direction in the comparison and assessment of the effectiveness and safety of Molnupiravir in the treatment of COVID-19 versus standard care alone. Two key phase III trials are built upon a voluntary licensing agreement between Hetero and MSD for the investigation, manufacture, and distribution of Molnupiravir in the treatment of COVID-19. Each of the two trials were planning to recruit 1218 patients for the efficacy assessment. On July 9, 2021, Hetero published an interim report for phase III trial on 741 mild COVID-19 patients (HETERO, 2021). Interestingly, the report presented very promising outcomes especially regarding the need for hospitalization (Table 3).

**Table 3:** Interim report for phase III trial on 741 mild COVID-19 patients published by Hetero.

Significant early clinical improvement in Molnupiravir treatment group in comparison to standard care on days 5, 10 and 14 with 2-point reduction in WHO Clinical Progression Scale		
**Day**	**Molnupiravir vs Standard Care**	**P value**
Day 5	63.43% vs 22.33%	p=<0.0001
Day 10	78.96% vs 49.49%	p=<0.0001
Day 14	81.55% vs 73.22%	p = 0.0150

Clinical improvement median time in Molnupiravir was at 8 days while Standard of Care was at 12 days (p = 0.0001)		
Less hospitalization was observed in Molnupiravir group vs SOC over 14 days (7 (1.89%) vs 23 (6.22%), p = 0.0027)		
Negativity of SARS CoV-2 RT-PCR was early observed in Molnupiravir group in comparison to standard of care		

**Day**	**Molnupiravir vs Standard Care**	**P value**
Day 5	77.35% vs 26.07%	p=<0.0001
Day 10	94.03% vs 57.20%	p=<0.0001
Day 14	97.01% vs 85.21%	p=<0.0001
No mortality was reported in both groups		

## 9 Discussion

Molnupiravir showed potent antiviral activity against SARS-CoV-2 virus (Kabinger et al., 2021, Zhou et al., 2021b). The drug is also expected to be active against the Omicron VOC, although in vitro and in vivo data are currently limited (Vangeel et al., 2022). Nonetheless, its mutagenic antiviral activity speculated a risk for being metabolized and incorporated into the human DNA, leading to consequent mutations. Furthermore, the mutagenicity of Molnupiravir was investigated in a study that utilized a rodent mutagenicity assay; the study showed no evidence for mutagenicity. Based on the conducted research on genotoxicity of the drug, the FDA concluded that Molnupiravir has a low risk for genotoxicity on a short treatment course of 5 days (FDA, 2021a). Upon completion of phase III clinical trial [MOVe-OUT] on 1433 patients, the drug was granted EUA from the FDA on December 23rd, 2021, for treatment of mild to moderate COVID-19 patients with at least one risk factor for progression and for whom the other FDA approved treatment modalities are not available or accessible. Of note, the FDA required the manufacturer to conduct a thorough investigation to justify the difference in efficacy observed upon interim versus the final all sample analysis. The authorization letter stated that Molnupiravir should not be indicated for pregnant women based on the fetal toxicity reported in the preclinical studies. Also, the letter underlined that Molnupiravir may cause AEs for infants through breastfeeding. The common AEs of molnupiravir are diarrhea and nausea, and the FDA stated no drug-drug interaction was identified (FDA, 2022). The drug was found promising so that 27 generic manufacturers signed agreements to produce Molnupiravir to be supplied for 105 low- and-middle-income countries (The Medicines Patent Pool, 2022).

The FDA guidelines set challenging clinical endpoints for the anti-viral drugs investigated to treat mild to moderate COVID-19 infections. These drugs should exhibit significant effect on the major clinical outcomes like all-cause mortality and need for hospitalization (FDA, 2021b). Thus, they are challenged with a relatively small effect size; as probability for disease progression to cause hospitalization or mortality doesn’t exceed 5–10% among mild to moderate cases (Mahendra et al., 2021). Till mid of 2021, the NIH COVID-19 treatment guideline was stating “No therapy has been proven to be beneficial in outpatients with mild to moderate COVID-19 patients who are not at high risk for disease progression”. At this time, only Remdesivir was granted EUA by FDA since it was reported to reduce recovery time in patients suffering from severe COVID-19 infections. However, the application of Remdesivir was limited for that it’s only administered via intravenous route in a hospital setting. Currently, despite there is an increasing list of antiviral drugs for treatment of mild to moderate cases with risk for progression, no therapy has been yet proven to treat mild to moderate cases with no risk for progression. This may be explained by the very low risk for progression for this population.

Overall, despite the proof that oral Molnupiravir is effective for the treatment of COVID-19, without evident safety concerns, when initiated within 5 days after the onset of signs or symptoms in non-hospitalized unvaccinated adults, the FDA and NIH COVID-19 treatment guideline both recommended using Molnupiravir only when Ritonavir-boosted Nirmatrelvir, Sotrovimab, and Remdesivir are not available (NIH, 2022). Ritonavir-boosted Nirmatrelvir was also granted the FDA EUA on December 22, 2021 (FDA, 2021c), for treatment of mild to moderate cases with risk for progression. The EPIC-HR trial showed that starting Ritonavir-boosted Nirmatrelvir, within 5 days of symptom onset, in adults with mild to moderate COVID-19 reduced the risk of hospitalization/death by 89% through Day 28 (Hammond et al., 2022). This significant reduction in the primary efficacy endpoint was comparable to that reported for Sotrovimab (85% relative reduction) (Gupta et al., 2021) and Remdesivir (87% relative reduction) (Gottlieb et al., 2022), and greater than the finally reported efficacy for Molnupiravir (30% relative reduction) (Jayk Bernal et al., 2021).

Currently, Molnupiravir is being investigated for protection against COVID-19 infection in a multi-center clinical trial [MOVe-AHEAD, NCT04939428]; where the target population are subjects who live in a household with a person recently confirmed with COVID-19 infection. It also worth noting that Molnupiravir was tagged for successful combination with favipiravir in the clinical settings; anticipating a synergistic and more potent antiviral activity against COVID-19 (Abdelnabi et al., 2021b). These findings may be the basis for large clinical studies to test the efficacy of the proposed combination of Molnupiravir/Favipiravir for treatment of COVID-19.

## 10 Strengths and limitations

To the best of our knowledge, this is the first systematic review that thoroughly cover both preclinical and clinical studies on Molnupiravir as an antiviral therapy to treat COVID-19 infections. However, lack of data from clinical trials made it a qualitative review without a quantitative compiling of the clinical evidence through *meta*-analysis.

## 11 Conclusion

The conducted preclinical and clinical studies confirm the efficacy and safety of Molnupiravir as an antiviral therapy to treat mild to moderate COVID-19 infections with high risk for progression. Nonetheless, as for all repurposed antiviral drugs, more data from clinical trials is still required to determine its exact efficacy and tolerability; especially for special population. Molnupiravir is currently being investigated for the potential use in post-exposure prophylaxis against COVID-19 and tagged for possible combinations with other antivirals; which if found to be effective, it would be a new turning in the COVID-19 pandemic situation.

## Declaration of Competing Interest

The authors declare that they have no known competing financial interests or personal relationships that could have appeared to influence the work reported in this paper.
